# How to provide vascular control of splenic artery aneurysms? A case series

**DOI:** 10.1016/j.amsu.2020.08.035

**Published:** 2020-09-02

**Authors:** Vladimir Milosavljević, Mauricio Gonzalez-Urquijo, Boris Tadić, Nikola Grubor, Carlos Antonio Morales-Morales, Slavko Matic

**Affiliations:** a Gracia Medica Polyclinic, Koce Popovica Street, No. 9, 11000, Belgrade, Serbia; b Tecnologico de Monterrey, School of Medicine and Health Sciences, Ignacio Morones Prieto O 3000, Monterrey, 64710, Mexico; c Department for HPB Surgery, Clinic for Digestive Surgery, Clinical Centre of Serbia, Koste Todorovica Street,No. 6, 11000, Belgrade, Serbia

**Keywords:** Aneurysm, Case report, Laparoscopic splenectomy, Spleen, Splenic artery

## Abstract

**Background:**

Spleen artery aneurysm represents the most common visceral aneurysm and the third most common splanchnic aneurysm. Most patients have no symptoms and are diagnosed as a part of other diagnostic focuses and examinations. Greater prevalence and application of modern diagnostic and imaging procedures has resulted in greater detection of this disease.

**Results:**

We present two patients with splenic artery aneurysms localized in the splenic hilum, who auspiciously underwent laparoscopic splenectomies with the use of hem-o-lock clips in the vascular hilum without complications. Both postoperative courses were uneventful. At six months follow up, both patients are asymptomatic.

**Conclusion:**

These two cases showed that in addition to the numerous advantages of minimally invasive approaches for treating splenic arterial aneurysms, there is a possibility to improve laparoscopic technique in terms of safety and economic reasons by using hem - o - lock clips as a hemostatic technique for the vascular elements of the spleen hilum.

## Highlights

• Spleen artery aneurysm represents the most common visceral aneurysm.
• We present 2 patients with splenic artery aneurysms localized in the splenic hilum.
• Hem-o-lock clips can be safely used to secure the splenic hilium.

## 1 Introduction

Spleen artery aneurysm (SAA) represents a focal dilatation bigger than 50% of the artery lumen. Most often, the diameter of the splenic artery is about 50 mm. SAA represents the most common visceral aneurysm and the third most common splanchnic aneurysm [1,2].

Most patients with SAA have no symptoms and are diagnosed as a part of other diagnostic focuses and examinations. Higher representation and application of modern diagnostic imaging procedures, primarily computed tomography (CT) and magnetic resonance imaging (MRI), have resulted in a higher number of detected SAAs [3]. Symptomatic aneurysm of the splenic artery of any size, especially in pregnant women and women in the reproductive period, is an indication for surgical treatment because of the possible rupture and other complications that accompany this disease [3,4].

SAA treatment options include observation for those smaller than 20 mm, and surgical treatment, either by minimally invasive, endovascular, or conventional approach for those greater than 20 mm [5].

Herein, we report two patients who due to a spleen artery aneurysm localized in the spleen hilum, underwent laparoscopic splenectomy safely and effectively, without complications, with the use of hem-o-lock clips as a hemostatic technique for securing the vascular elements of the spleen hilum. The work has been reported in line with the PROCESS guidelines [6]. The work is registered in ClinicalTrials under ID number NCT04502147.

https://clinicaltrials.gov/show/NCT04502147

## 2 Case 1

**Fig. 1:**
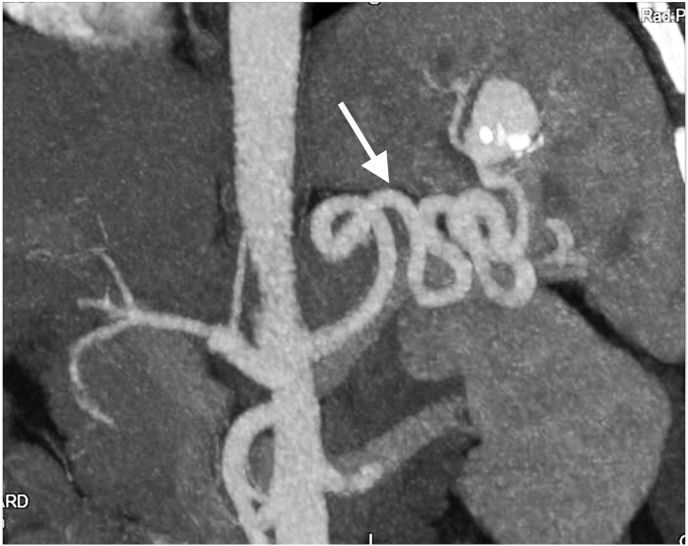
Coronal CT. White arrow showing splenic arterial aneurysm.

A 43-year-old patient, with no past medical history, was admitted to an academic institution for operative treatment of a splenic artery aneurysm diagnosed by a previous CT scan examination (Fig. 1). The examination was performed due to non-specific discomfort in the abdomen and occasional pain in the left subcostal margin. As a part of the additional diagnostics, an MRI was performed on which a tortuous splenic artery with an aneurysm in the spleen hilum, sized 27 × 20 mm, was diagnosed. Smaller aneurysms on the distal parts of the two lateral branches of the splenic artery were seen, as well as a 9 mm aneurysm on the right renal artery distal portion.

An attending surgeon performed the procedure. An artificial pneumoperitoneum by the usage of Veress needle with a pressure of 14 mmHg was performed. The ports were placed at typical sites for the operation. The abdomen's inspection verified a neat finding, so mobilization of the spleen was made, detecting an accessory spleen (AcS) of 1 cm, which was not seen on preoperative imaging. A decision to preserve the AcS was made, since the patient had no hematological spleen disease. A laparoscopic harmonic scalpel (Ethicon Endo-Surgery, Inc, Cincinnati, Ohio, USA) to release the spleen from its ligaments and the short gastric vessels was used. The splenic artery was extremely curved, in close contact with the body and the tail of the pancreas. Separation of the spleen from the splenic hilium was done placing hem-o-lock clips at the hilium. Instrumental destruction of the spleen was performed and sent to histopathological examination (HP). After removing the spleen, hemostasis was verified, and a drain was placed in left subphrenic space.

The drain was removed on the second postoperative day. The patient was discharged from the academic institution on the third postoperative day with antibiotic therapy and immunization required, following current literature guidelines for the prevention of post-splenectomy infections.

The definitive HP finding showed that the splenic tissue had preserved histomorphology, and the splenic artery fragment was aneurysmally altered, sclerosing, with occasional calcifications.

## 3 Case 2

**Fig. 2:**
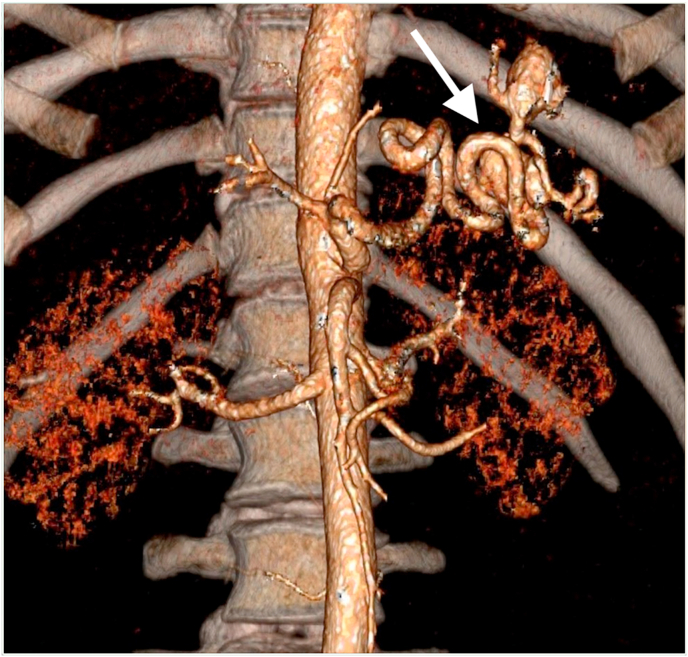
Coronal CT reconstruction. White arrow showing splenic arterial aneurysm.

A 41-year-old patient, with no relevant past medical history, was diagnosed with splenic artery enlargement on a routine ultrasound examination. As part of the additional diagnostics, a CT scan of the abdomen with angiography was performed. A saccular aneurysm of the splenic artery measuring 24 × 17 mm was diagnosed in the splenic hilum (Fig. 2). Within the aneurismatic dilatation, a wall thrombus sized 3 mm was detected.

**Fig. 3:**
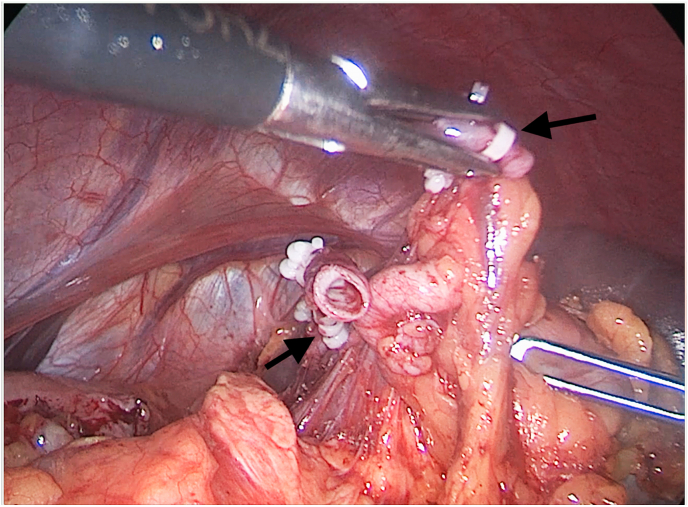
Laparoscopic view. Black arrows showing hem-o-locks after splenic artery section.

The patient was electively admitted to an academic institution for treating his splenic aneurysm. A general surgery attending performed the procedure. After performing an artificial pneumoperitoneum and placing the working ports, inspection of the abdominal cavity was carried out, identifying a splenic artery aneurysm at the hilium. Mobilization of the spleen releasing it from its ligaments, and gastric short vessels was completed. After dissecting the splenic artery, proximally to the aneurysm, two hem-o-lock clips were placed securing the aneurysm (Fig. 3). The splenic vein was treated the same way. After taking care of the vascular elements of the hilum, the spleen was separated entirely from the surrounding structures, placed in an endo-bag, and removed from the abdominal cavity in fragments after instrumental destruction. The spleen with the aneurysm was sent for the HP verification.

A definitive HP finding indicated that splenic tissue had preserved histomorphology, along with a splenic artery saccular aneurysm with a thrombus on the arterial wall and focal calcifications.

The postoperative course was uneventfully, and the abdominal drain was removed on the second postoperative day. The patient was discharged from the clinic on the third postoperative day with prescribed antibiotic prophylaxis and postoperative immunization.

In both cases, both patients were monitored by performing abdominal ultrasounds, one and three months after their initial surgeries, respectively. At six months follow up, an MRI was ordered on both patients without any abnormal findings. Patients are currently in follow up without any complication.

## 4 Discussion

The etiologic factors associated with the onset of SAA are atherosclerosis, port hypertension, age, sex, pregnancy, trauma, iatrogenic injury, Marfan's, and Ehler-Danlos syndrome [7]. According to data from the current literature, the prevalence of SAA in the general population is less than 1%, since in most cases, the aneurysm of the splenic artery is asymptomatic, and it is infrequently detected [8].

When there is expressed symptomatology, it is presented as pain in the epigastrium with penetration to the left shoulder, bleeding from the upper gastrointestinal tract, bleeding from the Wirsung duct, hemobilia, among others less common [1].

CT represents an ideal diagnostic modality for SAA detection, the evaluation of anatomical relationships with surrounding structures, and the evaluation of surgical treatment. MRI is an alternative method for CT. Endoscopic ultrasound examination may be useful in distinguishing SAA from other similar lesions such as pancreatic pseudocysts. However, contrasting CT angiography is the most specific and most sensitive method [3,9].

The laparoscopic approach can be used for diagnostic and therapeutic purposes, especially in detecting AcS, as we have shown in the first case. Furthermore, better detection of AcS by using a laparoscopic approach, over the preoperative imaging diagnostic, has been previously demonstrated [10].

For aneurysms bigger than 20 mm in diameter, due to an increased risk of rupture, surgical treatment is recommended in patients with expressed symptomatology, in women of reproductive age, and patients with cirrhosis [11]. In our patients, the diameter of the aneurysm was bigger than 20 mm; therefore, we decided to perform surgical treatment in both cases.

Treatment modalities include conventional or laparoscopic splenectomy (LS) or endovascular treatment of the splenic artery. Splenectomy or organ-preserving techniques, or resection of the aneurysm with arterial reconstruction might be applied, depending on the location and size of the aneurysm and the characteristics of the patients [5,12].

Endovascular treatment can be employed for proximal aneurysm localizations, or for treating pseudoaneurysms. The main contraindications for this approach are distal or hilar localization of aneurysms, poorly developed collateral vessels, or high-risk patients [13].

LS is the method of choice when the aneurysm is localized in or nearby the hilum. Depending on the size of the aneurysm, it may sometimes be necessary to perform a distal pancreatectomy because of the closeness of the aneurysm and the tail of the pancreas. The benefit of LS is also given in pregnant women, given to all the benefits it has in comparison with open surgery, with less likelihood of preterm delivery [14,15].

## 5 Conclusion

Laparoscopic splenectomy is an effective and easily applicable modality for treating SAA localized in the spleen or nearby the hilum. These two cases show that in addition to numerous advantages of a minimally invasive approach, there is a possibility to improve laparoscopic technique in terms of safety and economic reasons by using hem-o-lock clips as a hemostatic technique for the vascular elements of the spleen hilum. We believe by using this technique, there can be minor intraoperative bleeding and less risk of pancreatic injury. In addition, hem-o-lock clips are cheaper than endovascular staplers, so their use can reduce the overall surgery treatment cost. Further prospective studies are needed comparing different approaches for treating this diseases.

## Ethical approval

Ethics Committee of the Clinical Center of Serbia.

DECISION: We approve the academic clinical work “How to provide vascular control of splenic artery aneurysm? A Case Series” to be conducted.

No. 168/7.

Date: May 26, 2020.

## Sources of funding

This research received no external funding.

## Author contribution

Conceptualization, V.M.; M.G.U; B.T; Writing-review and editing, B.T; V.M.; N.G.; Literature review, B.T.; C.M.M; M.U.; Writing draft, V.M.; M.G.U.G.; Supervision, S.M.

## Registration of research studies

1. Name of the registry: How to Provide Vascular Control of Splenic Artery Aneurysm? A Case Series
2. Unique Identifying number or registration ID: NCT04502147
3. Hyperlink to your specific registration (must be publicly accessible and will be checked): https://clinicaltrials.gov/show/NCT04502147

## Guarantor

Mauricio Gonzalez Urquijo.

## Consent

Written informed consent was obtained from the two patients for publication of this case report and accompanying images. A copy of the written consent is available for review by the Editor-in-Chief of this journal upon request.

## Provenance and peer review

Not commissioned, externally reviewed.

## Declaration of competing interest

The authors declare no conflict of interest.
